# Nerve endings reveal hidden diversity in the skin

**DOI:** 10.7554/eLife.00352

**Published:** 2012-12-18

**Authors:** Eric E Turner

**Affiliations:** Center for Integrative Brain Research, Seattle Children's Research Institute, Seattle, USAeric.turner@seattlechildrens.org

**Keywords:** skin, neuronal morphology, sparse labeling, receptive field, Brn3a, Mouse

## Abstract

Experiments on transgenic mice have revealed that the morphologies of sensory neurons in the skin of mice are more complex and diverse than expected.

**Related research article** Wu H, Williams J, Nathans J. 2012. Morphologic diversity of cutaneous sensory afferents revealed by genetically directed sparse labeling. *eLife* **1**:e00181 doi: 10.7554/eLife.00181

**Image** Nerve endings associated with hair follicles in the skin of a mouse

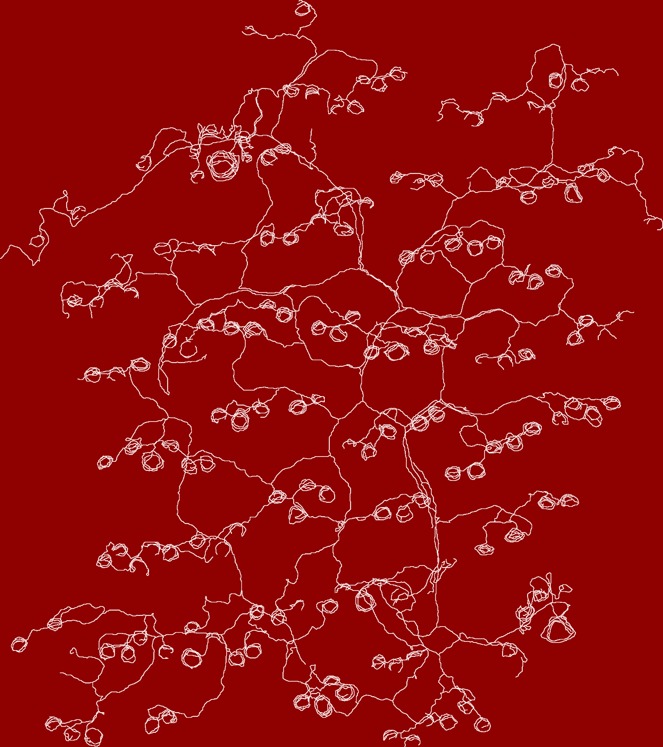

Sensory neurons are the brain's portal to the external world. Four of the five traditional senses, the ‘special senses’ of vision, scent, hearing and taste, are conveyed by discrete sense organs that contain a few types of highly specialized signal transducing cells, such as rods and cones in the retina, or cochlear hair cells in the ear. However the sense of touch, which is conveyed by general somatic sensory neurons, is much less well defined. These neurons reside in discrete ganglia that lie peripheral to the brainstem and spinal cord, including the trigeminal ganglia that receive signals from the face and head, and the dorsal root ganglia that serve the trunk and limbs. Traditionally, somatic sensory neurons have been divided into three broad subtypes: nociceptors (for sensing pain), mechanoreceptors (for sensing touch), and proprioceptors (which sense body position). Nociceptors and mechanoreceptors terminate in the skin, whereas proprioceptors terminate in muscles and tendons.

It has long been recognized that nerve endings in the skin display a diverse range of forms, but prior studies have generally used histological methods in tissue sections that do not reveal the complete morphology of each neuronal axon. Now, writing in *eLife*, Hao Wu, John Williams and Jeremy Nathans of Johns Hopkins University report the results of experiments that involve some very modern transgenic tricks, yet evoke the studies of neuronal morphology in the early 20^th^ century—using methods introduced by Golgi and perfected by Ramón Y Cajal—that first revealed the complex architecture of single neurons (see Figure 1). The results of the Johns Hopkins experiment are largely descriptive in nature, and we might assume that the results reported are already buried somewhere in the literature, but they are not. Thus we are reminded that our knowledge of even well-studied experimental systems is still very fragmentary.

**Figure 1.**
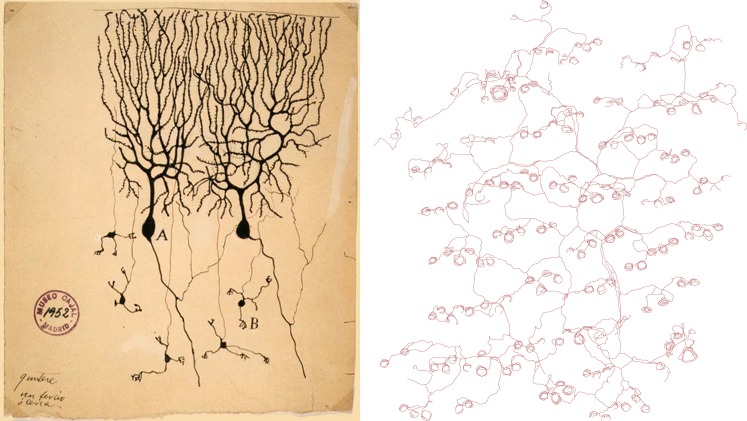
Drawings of neurons and nerve endings made more than a century apart. The drawing on the left was made by Santiago Ramón Y Cajal in 1899 and shows Purkinje cells in the cerebellum of a pigeon. The cells were stained with potassium dichromate and silver nitrate. The trace on the right shows nerve endings in the skin of a mouse. A combination of genetic and histochemical techniques were used to record the image from which the trace is taken (Wu et al., 2012).

IMAGE: INSTITUTO SANTIAGO RAMÓN Y CAJAL

This latest work was made possible by the Cre-Lox system—a widely-used approach in which a Cre recombinase enzyme is used to remove chromosomal DNA flanked by two genetically engineered loxP recognition sequences. Wu, Williams and Nathans used this method to excise a signal sequence blocking the expression of a histochemical marker gene which had been previously engineered into the chromosomal location of a transcription factor (Brn3a) that is important in the development of the sensory nervous system. A key feature of the experiments was the use of a form of the Cre enzyme that is translocated to the nucleus only in the presence of an estrogen-like drug, tamoxifen, so the probability that the marker gene is expressed should be related to tamoxifen concentration. By trial-and-error titration of the tamoxifen dose administered to pregnant mice bearing transgenic litters, it was possible to label a small number of discrete sensory neurons.

The Johns Hopkins researchers attempt to systematically categorize, for the first time, the complex axons of many individual sensory neurons with respect to their morphology, the number and density of their endings, and also their relationship to hair follicles, where most of the fibers terminate. They acknowledge that this must be a preliminary system, because only a subset of sensory neurons is sampled. One class of axon terminal may be consistent with fibers conveying itch sensation, but most of the endings conveying pain, pleasant and unpleasant temperatures, and chemical irritants are probably not revealed by the labeling method used. One particularly interesting class of labeled neuron possesses axons with C-shaped endings that only partly encircle hair follicles, and that terminate on a consistent side of the follicles arrayed across a large skin area. This structure may be especially suited to convey the direction of movement of tactile stimuli across the skin. Recently it has become possible to correlate some well-known markers of sensory subtypes with the terminal morphology and electrophysiological properties of mechanoreceptors (Li et al., 2011), and the function of most of the mechanoreceptors identified here await further physiological studies.

It will be interesting to see to what extent these diverse patterns of nerve endings are pre-ordained by gene regulatory programs during the developmental period when the axons are growing out from the sensory ganglia, and how much is adaptive. The overall gene regulatory cascade for the early specification of pain, touch and proprioceptive somatic sensory neurons is now fairly well understood (Liu and Ma, 2011). In mice lacking the transcription factors Islet1 and Brn3a (the gene locus used to target the reporter in this study), sensory neurons remain in a generic ‘ground state’ of differentiation and express few subtype specific markers (Dykes et al., 2011). Recent work has shown that the receptor tyrosine kinase cRet (Luo et al., 2009) and the transcription factor cMaf (Wende et al., 2012) are required for the development of some classes of mechanoreceptors. However, none of these developmental mechanisms comes close to offering an explanation for the diversity of sensory arbors observed by Wu, Williams and Nathans. If a large part of the diversity observed in the present study is genetically determined, then much the regulatory program of sensory differentiation is yet unknown.

A related question is whether the pattern of arborization will be similar in regions of skin with very different sensory properties. It is well known that the size of touch receptive fields differs widely between areas that are sparsely innervated, such as the trunk skin studied by the Johns Hopkins group, and those that are densely innervated, such as the face and fingertips. In areas with finer resolution of tactile stimuli, such as the distal limbs and face, the arbors should have smaller territories and/or have more densely packed endings. Also, since the majority of neurons described here innervate hair follicles, the glabrous skin of the hands and feet must necessarily have differently structured endings and arbors.

It is remarkable that in the brachial and lumbar regions, the receptive field of a single dorsal root ganglion may encompass a continuous area of skin from the mid-back to the tips of the digits (Takahashi et al., 2003). A single dorsal root ganglion must therefore contain at least three fundamental classes of sensory neuron, with remarkable morphological and functional diversity expressed in each class. The Johns Hopkins study is only a ‘first pass’ at a systematic description of this diversity, so it is possible that a truly remarkable range of neuronal form and function will ultimately be found within a single sensory ganglion that contains just a few thousand neurons.
